# Research hotspots and trends of hyperbaric oxygen therapy in stroke: bibliometrics and visual analysis

**DOI:** 10.4103/mgr.MEDGASRES-D-25-00063

**Published:** 2026-01-23

**Authors:** Lifen Lu, Lijia Zhao, Yujie Xie, Li Wang, Yuxiu Ji, Xi Luo, Chi Zhang

**Affiliations:** 1 Rehabilitation Medicine Department, The Affiliated Hospital of Southwest Medical University, Luzhou, Sichuan Province, China; 2 Rehabilitation Medicine and Engineering Key Laboratory of Luzhou, Department of Rehabilitation Medicine, Southwest Medical University, Luzhou, Sichuan Province, China

**Keywords:** bibliometrics, cerebral hemorrhage, cerebral infarction, hyperbaric oxygen, hyperbaric oxygen preconditioning, hyperbaric oxygen therapy, hyperbaric oxygenation, ischemic stroke, stroke, visual analysis

## Abstract

As a non-invasive and drug-free treatment method, hyperbaric oxygen therapy has been applied in cerebrovascular accidents to improve cerebral hypoxia, reduce ischemia–reperfusion injury following stroke and promote neural repair. However, its clinical efficacy remains controversial. This work aims to analyze the progress of research on the role of hyperbaric oxygen in stroke through bibliometric methods and takes relevant literature from the Web of Science core database from 2007 to December 31, 2024. The results showed that from 2007 to the present, the annual number of English publications in this field has shown a fluctuating but gradual upward trend, with the peak in publication volume occurring in 2015. China is in a leading position in this field, but there is still a lack of international cooperation and high-impact journal articles from Chinese institutions and researchers. The top five keywords were “hyperbaric oxygen” (84 occurrences), “stroke” (46 occurrences), “hyperbaric oxygen therapy” (45 occurrences), “focal cerebral ischemia” (27 occurrences), and “cerebral ischemia” (26 occurrences). A total of 13 major research areas were identified, including #0 stem cells, #1 hyperbaric oxygen, #2 perfusion, #3 EPR oximetry, #4 tPA, #5 rehabilitation, #6 acute stroke, #7 TBI, #8 stroke medicine, #9 hyperbaric oxygen preconditioning, #10 preconditioning, #11 ischemia–reperfusion injury, and #12 hyperbaric. At present, there are relatively few clinical trials registered on hyperbaric oxygen therapy for the treatment of stroke. Due to the fact that the effectiveness of hyperbaric oxygen therapy depends on the type and timing of stroke, there is currently no unified standard for the optimal timing and duration of hyperbaric oxygen therapy. Overall, hyperbaric oxygen therapy is safe and reliable, with most adverse reactions being mild and reversible. In summary, hyperbaric oxygen therapy has significant neuroprotective and functional recovery potential in stroke patients.

## Graphical abstract

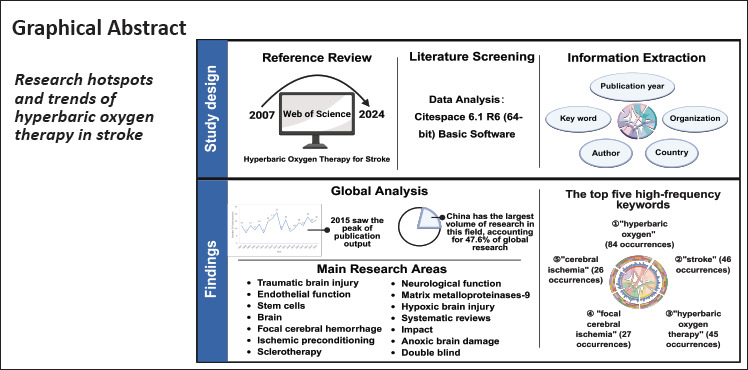

## Introduction

Stroke is the second leading cause of death worldwide and a major cause of disability.^1^ It is characterized by high morbidity, disability and mortality. According to the 2021 Global Burden of Disease Study, the estimated mortality rate is 44.^2^ deaths per 100,000 people, and approximately 50% of survivors experience varying degrees of disability. This imposes substantial economic and social burdens on patients’ families and society.^2^ Although intravenous thrombolysis and endovascular therapy have significantly improved the prognosis of acute stroke patients,^3^ long-term rehabilitation of neurological dysfunction caused by neurological deficits remains a clinical challenge.^4^ Therefore, new adjuvant treatment methods are urgently needed.

Hyperbaric oxygen (HBO) therapy (HBOT) is a medical treatment that involves the use of oxygen at an ambient pressure higher than 1 atmosphere absolute (ATA; 1 ATA = 101.325 kPa).^5^ Typically, the pressure is increased to 2.0–2.8 ATA for 90–120 minutes, with the number of treatments varying depending on the specific condition.^6^ As a non-pharmacological therapy, the mechanisms of HBOT primarily include improving cerebral hypoxia, reducing ischemia–reperfusion injury following stroke and promoting neural repair, thereby alleviating cognitive dysfunction and depression after stroke.^7^ Additionally, HBOT can reduce brain edema and intracranial pressure, improve cerebral circulation and create favorable conditions for the recovery of neurological function after stroke.^8^

Although HBOT has demonstrated potential in stroke treatment, its clinical efficacy remains controversial.^8^ Therefore, we employed literature visualization and bibliometric analysis methods to analyze and summarize research trends, hotspots and prospects of HBOT in stroke, aiming to provide a reference for the clinical application of HBOT in stroke treatment.

## Methods

### Data sources and retrieval strategies

The study data were extracted from the Web of Science Core Collection database. A systematic literature search was conducted for publications from January 2007 to December 2024, and the key English search terms included “hyperbaric oxygen” and “stroke.” The specific search query in the Web of Science Core Collection is shown in **Table 1**.

**Table 1:** Literature search results of hyperbaric oxygen therapy in stroke patients from the Web of Science Core Collection

	Search query	Result
1	TS=("hyperbaric oxygen "OR" hyperbaric oxygen therapy" OR "hyperbaric oxygen therapies" OR “HBO therapy”)	7642
2	TS=("stroke "OR" cerebral infarction" OR" cerebral hemorrhage "OR "TIA" OR "cerebral apoplexy" OR "cerebral infarction" OR "cerebral hemorrhage" OR "cerebrovascular accident")	359880
3	#1 AND #2	428

### Literature inclusion and exclusion criteria

The inclusion criteria were as follows: (1) journal articles related to HBOT for stroke; (2) article types limited to research articles and review articles; and (3) publications written in English.

The exclusion criteria were as follows: (1) duplicate publications and (2) articles unrelated to the research topic.

### Literature screening and data extraction

Two researchers (LW and XL) independently conducted the literature search and data extraction and cross-checked the results. If there were differences between the two researchers, the third researcher (YJ) solved the problem. The title and abstract were read first in the literature screening, and after excluding irrelevant literature, the full text was further read to determine whether the article was ultimately included. The data of the included studies were extracted, and the extracted information included authors, institutions, keywords, publication years and countries.

### Statistical analysis

CiteSpace 6.1. R6 (64 bit) software (Drexel University) was used to visualize the scientific knowledge map,^9^,^10^ so as to present the development process and structural relationship of scientific knowledge. The specific operation steps are as follows: First, the documents included in the Web of Science Core Collection database were set to a full record format in plain text format with cited references. The txt file named “download_txt” was exported and imported into CiteSpace software to remove duplicate documents. Next, the parameters were set, the time span covered 2007–2024 with 1-year time slices, and the node selection method employed the g index (k = 25, Top *N* = 50). For pruning, we selected the “Pruning sliced networks” option while keeping other parameters at their default settings and then analyzed keywords, countries, institutions, authors, co-cited references and other nodes individually.

## Results

### Search results

**Figure 1:**
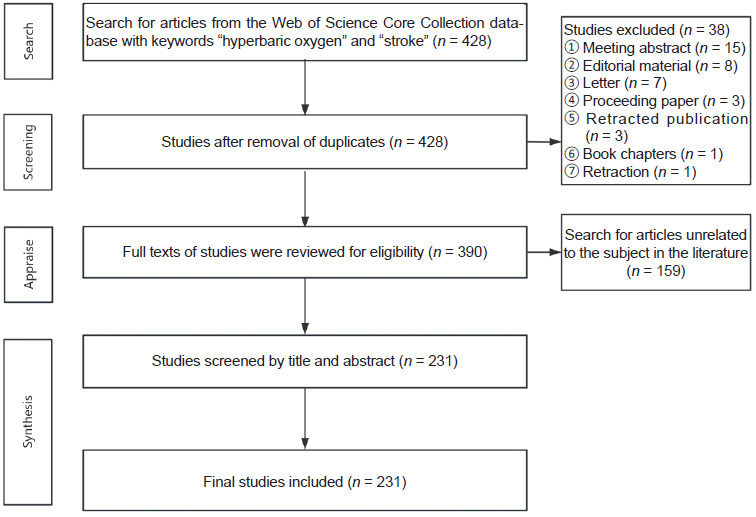
Flow chart of the literature screening process.

The initial search identified 428 articles, with 390 journal articles retained after applying the inclusion and exclusion criteria. Then, we further screened titles and abstracts, excluded 159 articles irrelevant to the research subject, and finally included 231 relevant studies. The literature selection process is detailed in **Figure 1**.

### The total publications, countries and institutions

**Figure 2:**
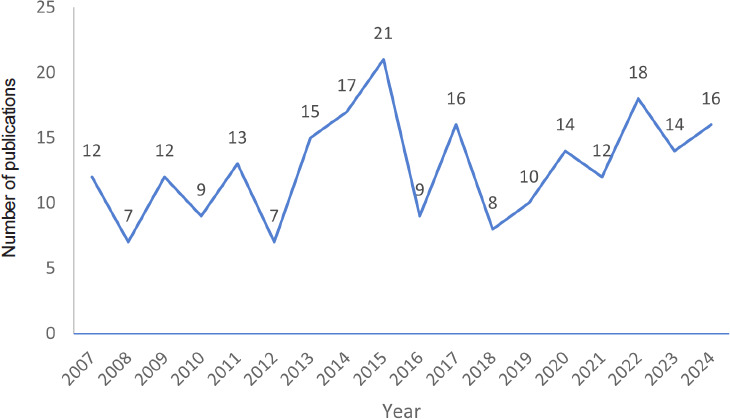
The annual number of publications on hyperbaric oxygen therapy for stroke from 2007 to 2024.

Changes in the number of publications over time are an important indicator for measuring development in this field. As shown in **Figure 2**, from the perspective of annual publication trends, the field demonstrated a pattern of slow but fluctuating growth, with the number of publications reaching its peak in 2015. From 2007 to 2024, global related research will continue to develop, but the growth rate will be relatively stable.

By 2024, China (including Taiwan Province) ranked first globally with 110 publications, accounting for 47.6% of the total publications, followed by the United States (58 publications), Germany (20 publications), Israel (14 publications) and Australia (7 publications). This distribution indicates that research activity in this field is significantly more prominent in China than in other countries, with Europe and America following (**Table 2**).

**Table 2:** Top 10 countries in terms of the number of publications on hyperbaric oxygen therapy in stroke patients

Rank	Country	Count	Centrality	Percentage
1	China	110	0.19	47.6
2	USA	58	0.37	25.1
3	Germany	20	0.23	8.7
4	Israel	14	0.13	6
5	Australia	7	0	3
6	UK	6	0.2	2.5
7	Canada	5	0	2.2
8	Poland	4	0.07	1.7
9	South Korea	4	0	1.7
10	Turkey	3	0	1.3

The top three institutions with the highest number of publications in this field are Loma Linda University in the United States (14 publications), followed by the Sackler Faculty of Medicine in Israel and Tel Aviv University in Israel, each with 12 publications (**Table 3**).

**Table 3:** Top 10 institutions in terms of the number of publications on hyperbaric oxygen therapy in stroke patients

Rank	Institution	Count	Percentage	Country
1	Loma Linda University	14	6.06	USA
2	Sackler Faculty of Medicine	12	5.19	Israel
3	Tel Aviv University	12	5.19	Israel
4	Air Force Military Medical University	10	4.33	China
5	Shamir Medical Center (Assaf Harofeh)	10	4.33	Israel
6	Chi Mei Hospital	8	3.46	China
7	Harvard University	6	2.60	USA
8	University of New South Wales Sydney	6	2.60	Australia
9	Massachusetts General Hospital	6	2.60	USA
10	Leipzig University	6	2.60	Germany

### Author collaboration

A total of 1086 authors contributed to relevant publications, with the five most prolific authors being Shai Efrati, Amir Hadanny, Ching-Ping Chang, Hailong Dong, and Michael H. Bennett (**Table 4**).

**Table 4:** Top 10 authors of publications related to hyperbaric oxygen therapy in stroke

Rank	Author	Count	Total number of citations	Average number of citations	County	H-index
1	Shai Efrati	9	8201	42.49	Israel	35
2	Amir Hadanny	5	1334	16.68	Israel	20
3	Ching-Ping Chang	5	2875	19.69	China	31
4	Hailong Dong	4	6081	28.55	China	42
5	Michael H Bennett	4	1945	32.97	Australia	23
6	C Hobohm	4	6187	71.11	Germany	34
7	Christopher French	4	18784	61.19	UK	67
8	Eshel Ben-jacob	4	11299	57.65	USA	60
9	Yair Bechor	4	985	32.83	Israel	15
10	Zongping Fang	4	663	15.79	China	13

**Figure 3:**
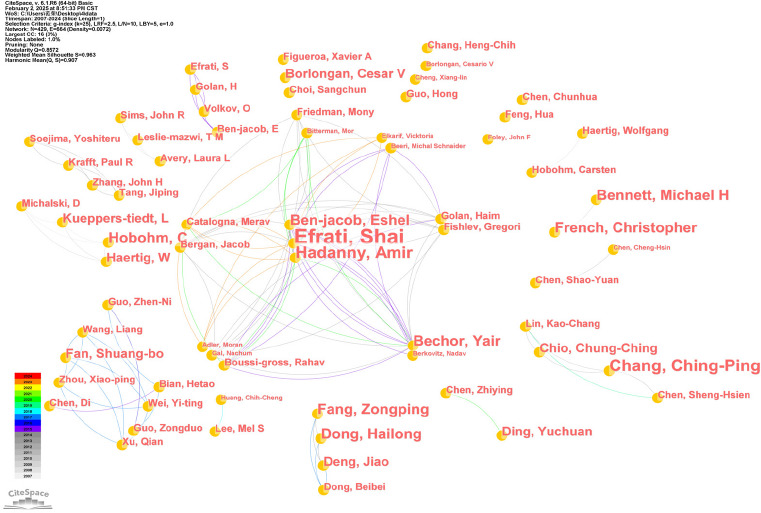
Author cooperation network for hyperbaric oxygen therapy for stroke. The font size represents the contribution rate of the authors, the lines between nodes represent the cooperative relationships between the authors, and the thickness and color of the lines represent the degree of cooperation and the time of appearance of the research results.

The author collaboration network reflects the collaborative relationships between the core author groups in the field. This study generated an author cooperation network map based on CiteSpace’s cooperation network analysis. As shown in **Figure 3**, the number of scholars engaged in HBOT for stroke is relatively small (429 nodes, 664 connections, network density 0.0072). While some scholars have established relatively stable collaborative groups, the overall collaboration network demonstrates limited connectivity among most authors, and there is no significant extended cooperation.

### Keywords

**Figure 4:**
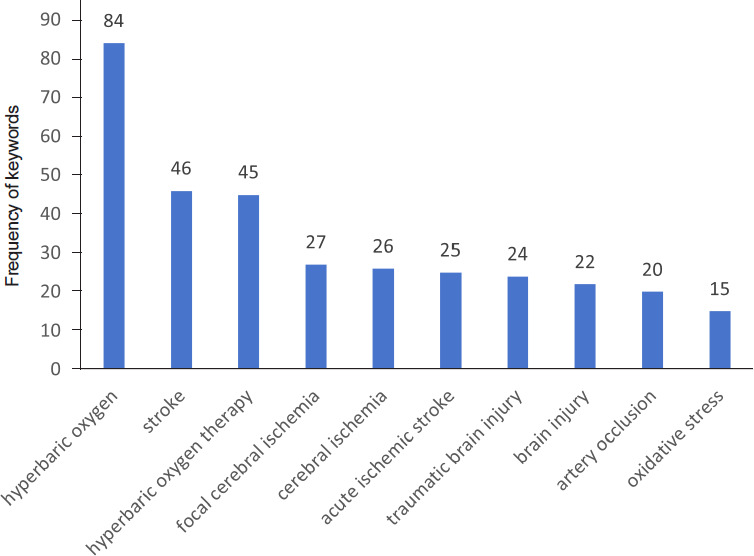
Top 10 keywords in the field of hyperbaric oxygen therapy for stroke.

Keywords reflect the central concept of an article and serve as a high-level summary of the article’s topic. High-frequency keyword analysis revealed that the top five keywords were hyperbaric oxygen (84 times), stroke (46 times), hyperbaric oxygen therapy (45 times), focal cerebral ischemia (27 times), and cerebral ischemia (26 times), as illustrated in **Figure 4**.

**Figure 5:**
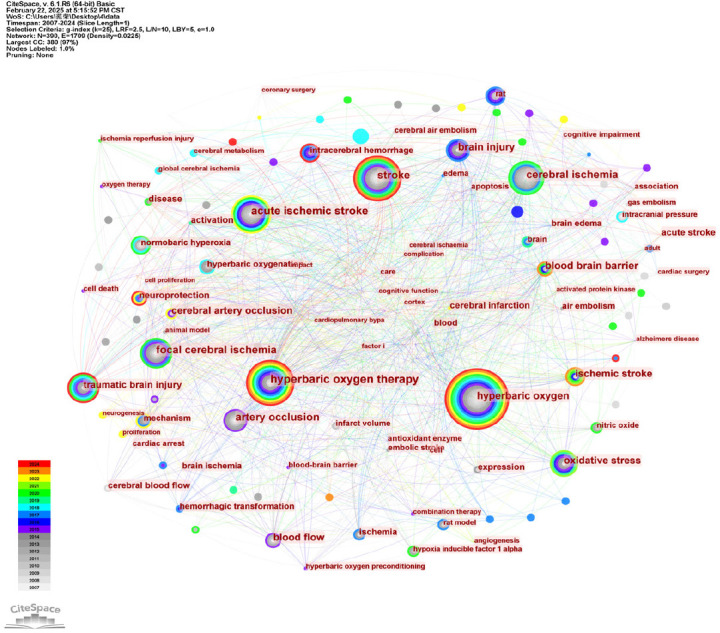
Co-occurrence map of keywords related to hyperbaric oxygen therapy for stroke. The font size indicates the frequency of keywords, the color change of the lines between keywords represents the connections established at different times, and the thickness and density of the lines indicate the strength of keyword co-occurrence.

The keyword co-occurrence network map of HBOT for stroke was obtained by setting “keyword” as the running node in CiteSpace (**Figure 5**). The map consists of 390 nodes and 1709 connections, with a network density of 0.0225.

**Figure 6:**
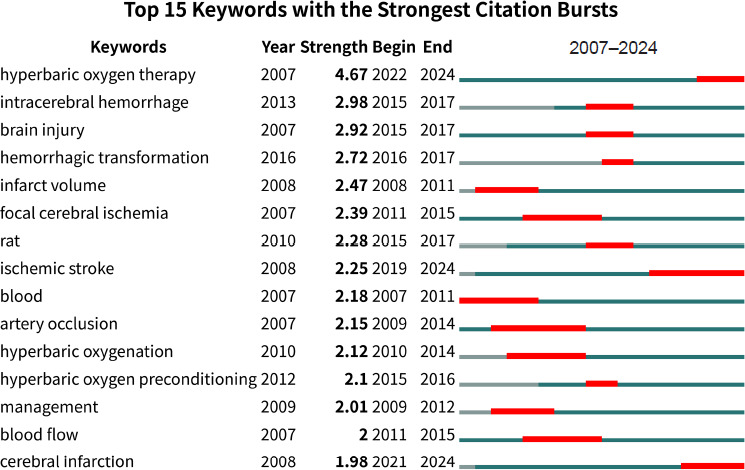
Emergence diagram of keywords related to hyperbaric oxygen therapy for stroke. The red portion represents the research frontiers at that time. The larger the burst is, the faster the growth rate and the higher the importance of the research.

**Figure 6** displays the top 15 keywords with the strongest bursts of English keywords. For example, from 2021 to 2024, cerebral infarction, HBOT, and ischemic stroke were identified as research frontiers and may become research hotspots in the future.

**Figure 7:**
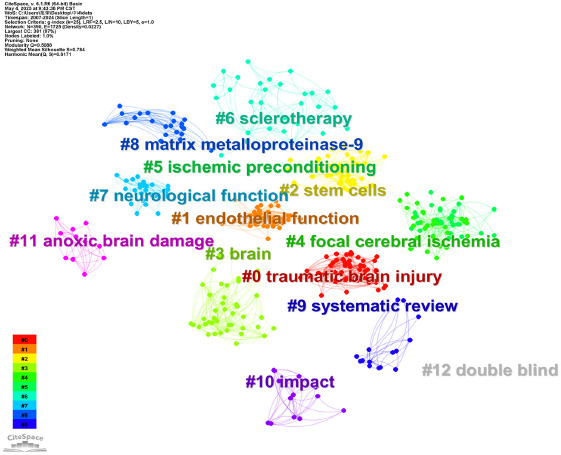
Keyword cluster diagram of hyperbaric oxygen therapy for stroke. The keyword co-occurrence network is clustered into irregular regions; each region corresponds to a label. The smaller the number is, the more keywords are included in the cluster.

CiteSpace software was employed to generate keyword cluster maps (**Figure 7**). The cluster analysis revealed 13 pivotal research directions in this field (silhouette = 0.784). These research directions are as follows: #0 traumatic brain injury; #1 endothelial function; #2 stem cells; #3 brain; #4 focal cerebral hemorrhage; #5 ischemic preconditioning; #6 sclerotherapy; #7 neurological function; #8 matrix metalloproteinases-9; #9 systematic reviews; #10 impact; #11 anoxic brain damage; #12 double-blind. Based on the keyword cluster map, we further obtained the trend of the number of publications in different research directions from 2007 to 2024.

**Figure 8:**
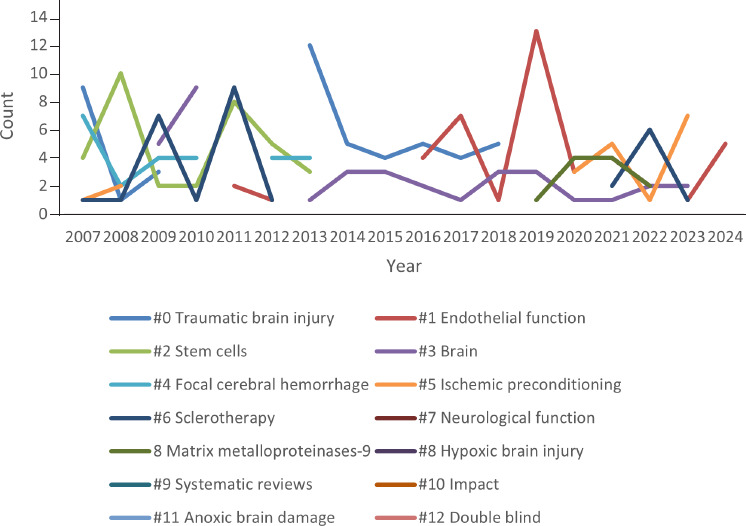
Trends in publications in different research directions on hyperbaric oxygen therapy for stroke.

The number of publications in the research direction of #0 traumatic brain injury was the largest, demonstrating a fluctuating publication trend. Research has shown a marked decline over the past 5 years. Currently, #5 Ischemic preconditioning has emerged as the most active research focus, which may be attributed to animal studies demonstrating that “ischemic preconditioning” reduces cerebral infarction volume^11^ and exerts a neuroprotective effect.^12^ However, clinical translation is still limited (**Figure 8**).

### Literature cocitation

**Figure 9:**
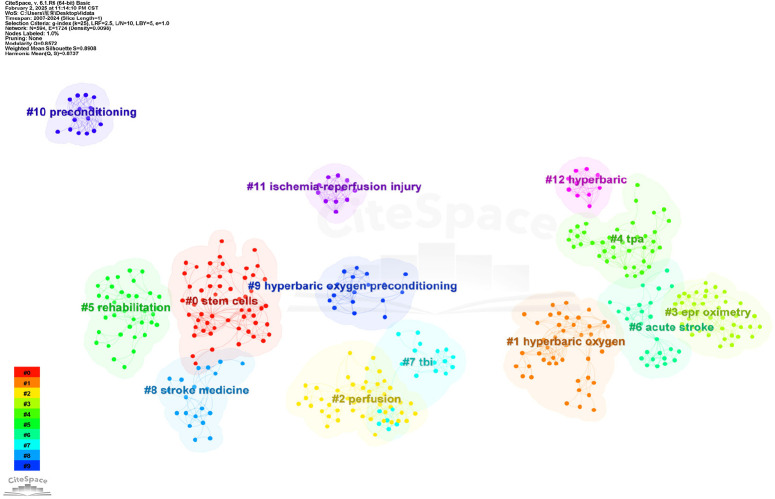
Co-citation map of the literature related to hyperbaric oxygen therapy for stroke. The block color of each cluster in the figure indicates the year when the co-citation relationship first appeared within that cluster. The citation timeline spans from left to right, representing from 2007 to 2024. The blue blocks denote the earliest publication year (2007), and the red blocks signify the most recent publication year (2024). Nodes represent cited articles identified by the first author’s name and publication year.

Co-citation analysis is a common method in bibliometric analysis. CiteSpace was used to generate the literature co-citation network (**Figure 9**). The co-citation analysis identified 13 major research clusters, representing primary research directions in the cited literature: #0 stem cells, #1 hyperbaric oxygen, #2 perfusion, #3 EPR (electron paramagnetic resonance) oximetry, #4 tPA (tissue plasminogen activator), #5 rehabilitation, #6 acute stroke, #7 TBI (traumatic brain injury), #8 stroke medicine, #9 hyperbaric oxygen preconditioning, #10 preconditioning, #11 ischemia–reperfusion injury, and #12 hyperbaric oxygen therapy.

A retrospective analysis by Hadanny et al.^7^ demonstrated that HBOT considerably improved cognitive function even in the late stages of stroke, and the selection of patients for HBOT after stroke should be based on functional analysis and baseline cognitive scores rather than on stroke type, lesion site or lesion side. This study is the most cited study in the literature (14 citations). A randomized prospective trial by Efrati et al.^13^ found that HBOT improved neurological function in patients with advanced stroke. This study revealed significant differences in brain anatomy and physiology in certain regions, where neuroplasticity can still be activated long after the injury has occurred. Another retrospective study by Helms et al.^14^ found that HBOT had a positive effect on the treatment of cerebral ischemia. However, the clinical effectiveness of HBOT for treating cerebral ischemia remains uncertain. Li et al.^15^ investigated the protective effect of HBO preconditioning on cerebral ischemia–reperfusion injury in rats through the inhibition of apoptosis through the mitochondrial pathway. This study revealed that HBO preconditioning could reduce cerebral edema, decrease infarct volume and promote the recovery of neurological function. Matchett et al.^16^ reviewed that HBOT has been shown to improve brain injury in various animal models, including focal cerebral ischemia, global cerebral ischemia, neonatal hypoxic–ischemia and subarachnoid hemorrhage. Singhal^17^ reviewed the historical application of oxygen therapy in stroke, the mechanism of HBOT, and findings from animal experiments and clinical research on hyperoxic interventions for cerebrovascular diseases. The results showed that oxygen therapy has neuroprotective potential and could save ischemic brain tissue and prolong the time window of treatment. Cheng et al.^18^ found that HBO preconditioning reduced cyclooxygenase-2 expression and provided cerebral protection after global ischemia. The administration of a cyclooxygenase-2 inhibitor with HBOT before ischemia abolished the preconditioning effect, indicating that cyclooxygenase-2 is a mediator of HBO preconditioning in the ischemic brain. Veltkamp et al.^19^ confirmed that HBOT could effectively reduce the permeability of the blood–brain barrier in ischemic brain tissue, significantly mitigate blood–brain barrier damage, and reduce vasogenic edema in T2-weighted images and tissue sections. These findings suggest that HBOT has a protective effect on blood–brain barrier damage and edema following transient focal cerebral ischemia. Beynon et al.^20^ conducted a rat experiment in which the infarct volume was 31% lower in the HBOT group than in the air group and 23% lower in the HBOT group than in the normobaric oxygen group. These findings suggest that HBO has greater clinical potential in mitigating cerebral ischemic injury. Eschenfelder et al.^21^ revealed that oxygen exerts a neuroprotective effect on acute transient focal cerebral ischemia and that this effect is dose dependent. The study confirmed that HBOT was superior to normobaric oxygen in terms of primary outcome measures, such as infarct volume reduction and clinical outcome improvement.

## Discussion

### Study results

This review systematically analyzed the literature related to HBOT in stroke patients. English-language publications on HBOT in the field of stroke have shown a slow but fluctuating increase in recent years. This trend indicates that a growing number of researchers are paying increasing attention to the development of HBOT in stroke treatment. China is a leader in this field, with the largest number of published studies globally. However, although many studies on HBO for stroke treatment have been published in China, Chinese institutions and researchers remain underrepresented in international research collaborations and high-impact journals. This may be related to the limited global academic influence of Chinese researchers in this field.

The frontier topics of research can be represented through keywords with citation bursts. This study analyzes the research frontiers and main fields of the HBOT-related literature on stroke via two keywords. HBOT is an emerging keyword, highlighting the significance of this treatment in stroke management. Since it was first documented in 1662,^22^ HBOT has been widely applied in the treatment of various diseases, and its application in the field of stroke has garnered increasing attention since 2007. There are currently few clinical trials on HBOT for stroke treatment, with only 13 registered trials related to this approach (ClinicalTrials.gov, last accessed on March 1, 2025). Therefore, we systematically searched the Web of Science database for clinical trials investigating HBOT in stroke patients. After excluding articles unrelated to the topic and retracted publications, a total of 14 relevant journal articles were identified, as detailed in **Table 5**.^13^,^23^,^24^,^25^,^26^,^27^,^28^,^29^,^30^,^31^,^32^,^33^,^34^,^35^

**Table 5:** Clinical trials related to HBOT in stroke patients

Study	Year	Country	Age	Sample size	HBOT intervention	Therapeutic time window	Adverse effect	Main conclusion
Harrison et al.^23^	2024	Canada	16–36 yr old	136	2.0 ATA with 100% oxygen, 90 min per session, 5 d a week for 40 sessions.	6–36 mon after onset	NR	Current evidence does not support the application of HBOT in chronic stroke.
Yuan et al.^24^	2022	China	50 to 77 yr old	118	1.48 ATA, the oxygen concentration was unknown, 60 min per session, once daily, 10 sessions per course, 2–4 courses may be required based on individual patient conditions	NR	NR	HBOT enhances neurological recovery in stroke patients with depression, markedly alleviating depressive symptoms and improving sleep quality.
Schiavo et al.^25^	2020	Canada	Over 18 yr old	27	2.0 ATA with 100% oxygen, 90 min per session, 5 d a week, over 8 wk for 40 sessions)	3 to 48 mon post-stroke	Four patients experienced middle ear barotrauma and one patient reported chest pain.	HBOT-exercise combined with mental imagery therapy is a safe and feasible approach for treating chronic stroke.
Xu et al.^26^	2018	China	Adult	79	2.5 ATA with 100% oxygen, 60 min per session, for a total of 60 d; Stratified by the time of stroke onset (0–6 h, 6–12 h, or 12–24 h), patients were randomized to receive HBOT	Within 24 h after onset	Eight patients had otalgia and/or claustrophobia.	Early application of HBOT is safe and effective in improving long-term neurological outcomes, and the HBOT group demonstrated superior neurological results compared to the NBOT group.
Kulai and Kovalchuk.^27^	2018	Russia	Control group: 65 ± 1.6 yr HBOT group: 66 ± 1.2 yr	112	1.5 ATA, 45 min per session, starting from the onset of the condition and continuing for 10 d	Hyperacute phase of ischemic stroke	NR	Mexidol combined treatment with HBOT significantly alleviated the severity of neurological impairments and systemic inflammation.
Li et al.^28^	2017	China	18 to 75 yr old	565	Group A: 1.34 ATA with 21% oxygen, 60 min per session, once daily, 5 consecutive days per week (weekdays only), for 90 sessions. Group B: 2.0 ATA with 100% oxygen, 60 HBOT sessions; 60 min per session, once daily, 5 consecutive days per week (weekdays only). Group C: 2.0 ATA with 100% oxygen, 90 HBOT sessions; 60 min per session, once daily, 5 consecutive days per week (weekdays only). Group D: 1.5 ATA with 100% oxygen, 60 HBOT sessions; 60 min per session, once daily, 5 consecutive days per week (weekdays only). Group E: 1.5 ATA with 100% oxygen, 90 HBOT sessions; 60 min per session, once daily, 5 consecutive days per week (weekdays only).	Within 6 h of onset	Five patients occasionally experienced excessive sweating during HBOT, and some patients who underwent decompressive craniectomy developed sunken or swollen bone windows.	HBOT improved the functional prognosis of patients after acute intracerebral hemorrhage surgery. The therapeutic effect of 1.5 ATA is comparable to that of 2.0 ATA. and 1.5 ATA with 60 pressure exposures can be considered one of the optimal HBOT protocols.
Yan et al.^29^	2015	China	63 ± 8.1 yr old	90	2.0 ATA with 100% oxygen, 60 min per session, 5 times per week, with one course of treatment lasting 4 wk	Within 1 month of onset	NR	HBOT combined with fluoxetine demonstrates superior antidepressant efficacy compared to conventional monotherapy approaches.
Churchill et al.^30^	2013	USA	18 to 76 yr old	63	1.5 ATA with 100% oxygen, 40 min per session, for 60 treatments	Within 12 mon of onset	NR	HBOT demonstrated improvements in memory, attention, balance/coordination, endurance and sleep quality.
Efrati et al.^13^	2013	Israel	Over 18 yr old	74	2.0 ATA with 100% oxygen, 90 min per session, 5 d a week, over 2 mon for 40 sessions)	6 to 36 mon post-stroke	Six patients experienced mild to moderate middle ear barotrauma, and two patients had a mild seizure (these patients had a history of epilepsy before joining the study).	HBOT significantly enhances neurological function in stroke patients during the late chronic phase.
Xing et al.^31^	2007	China	NR	72	1.184 ATA with 100% oxygen, 60 min per session, once daily, 10 sessions per course, two courses were administered, with a 1-d break between courses	NR	NR	Acupuncture combined with HBOT can promote the recovery of sensory and motor functions in patients with cerebral infarction, and improve balance disorders.
Imai et al.^32^	2006	Japan	Control group: 73.7± 10.6 yr HBOT group: 74.9± 12.1 yr	201	2.0 ATA with 100% oxygen, 60 min per session, for 7 d	Within 48 h of onset	Five patients experienced temporary ear pain, three had temporary hypertension, one reported lower back pain, and one had temporary tachycardia.	HBOT combined with intravenous edaravone has shown effectiveness in acute embolic stroke.
Rusyniak et al.^33^	2003	USA	Over 18 yr old	33	2.5 ATA with 100% oxygen, 60 min per session, for 1 treatment. Stratified by the time of stroke onset (0–12 h, or 12–24 h), patients were randomized to receive HBOT	Within 24 h after onset	Two cases of patients reported ear pain and claustrophobia.	HBOT shows poor efficacy and potential harm in acute ischemic stroke.
Nighoghossian et al.^34^	1995	France	20 to 75 yr old	34	1.5 ATA with 100% oxygen, 40 min per session, for 10 treatments	Within 24 h after onset	Three patients suffered from myocardial infarction, deterioration of neurological conditions associated with ischemic injury and claustrophobia.	HBOT shows potential efficacy, while its adverse effects remain unclear.
Anderson et al.^35^	1991	USA	20 to 90 yr old	39	1.5 ATA with 100% oxygen, 60 min per session. The first HBOT session was conducted within 6 h after study enrollment, and continued every 8 h for 15 sessions.	Within 2 wk of onset	One patient with a history of psychiatric illness experienced an acute psychotic episode, and one case of atelectasis occurred.	The experiment was not completed as scheduled.

ATA: Atmosphere absolute; HBOT: hyperbaric oxygen therapy; NR: not reported in the original paper.

The available clinical data (**Table 5**) show that more than 1600 stroke patients have undergone HBOT, with approximately 85% showing improvement in clinical outcomes. HBOT effectively improves the neurological function of stroke patients,^13^,^24^,^26^ saves neurons in the ischemic penumbra by increasing the oxygen supply, reduces the infarct area and thus reduces the disability rate and mortality of patients.^17^,^20^,^21^,^36^,^37^ In terms of functional rehabilitation, HBOT has shown a wide range of therapeutic potential. HBOT also improves upper limb motor function,^25^ cognitive function,^7^ depression,^8^,^24^ sleep quality and memory in stroke patients.^24^

The second research frontier is ischemic stroke, which is caused by the occlusion of cerebral arteries and accounts for approximately 85% of all stroke cases.^38^ Studies have shown that both cerebral hemorrhage and cerebral ischemia result in tissue hypoxia and cellular damage.^39^ Hypoxia not only impedes angiogenesis but also leads to reduced neuronal activity and the formation of new synaptic connections. The brain has a highly dense vascular network, so it is crucial for repairing these regions with adequate oxygen supply.^13^,^39^

Currently, the primary treatments for stroke include thrombolysis, thrombectomy, nutritional nerve, dehydration to reduce intracranial pressure, rehabilitation, and intravascular drug therapy.^3^ The goal of treating stroke is to reduce neuronal cell death by recanalizing cerebral blood vessels and restoring blood flow.^40^ In acute ischemic stroke, certain tissues can be effectively salvaged through rapid restoration of blood flow. These tissues are referred to as the ischemic penumbra.^36^ A timely oxygen supply to the ischemic penumbra can induce vasoconstriction and reduce intracranial pressure,^32^ thereby effectively rescuing neurons and reducing infarct size.^36^

As shown in **Table 6**, the co-citation analysis of the literature reveals 13 major research areas, which represent the core research directions of the cited studies.^7^,^13^,^14^,^15^,^16^,^17^,^18^,^19^,^20^,^21^ These areas include stem cell therapy, HBOT, HBO preconditioning, ischemia–reperfusion injury, tissue plasminogen activator and rehabilitation therapy. The distribution of these research areas indicates that the current focus is primarily on treatment strategies for stroke and their related mechanisms. These research directions not only highlight the potential value of HBOT in stroke treatment but also provide important clues for further investigations into its mechanisms of action. Future studies could further explore the combined application of HBOT with other treatment approaches (such as stem cell therapy and rehabilitation training) to optimize comprehensive treatment strategies for stroke.

**Table 6:** Literature hotspots and visual analysis in the field of HBOT for stroke

Study	Count	Centrality	Main conclusion	Cluster ID
Hadanny et al.^7^	14	0.27	HBOT improves neurocognitive functions of post-stroke patients.	#0 stem cells
Efrati et al.^13^	12	0.01	HBOT improves the neurological function of patients with chronic late stroke.	#2 perfusion
Helms et al.^14^	11	0.03	The clinical usefulness of HBOT on cerebral ischemia is not yet certain.	#3 EPR oximetry
Li et al.^15^	11	0.06	HBO preconditioning reduces ischemia–reperfusion injury by inhibition of apoptosis via mitochondrial pathway in rat brain.	#1 hyperbaric oxygen
Matchett et al.^16^	11	0	Neuroprotective mechanisms of HBO in the treatment of cerebral ischemia.	#6 acute stroke
Singhal.^17^	11	0.01	This article reviews the history and current rationale for the use of oxygen therapy in stroke, the mechanism of HBOT, and the results of animal and human studies of hyperoxia in cerebrovascular diseases.	#3 EPR oximetry
Cheng et al.^18^	10	0.15	Cyclooxygenase-2 mediates HBO preconditioning in the rat model of transient global cerebral ischemia.	#9 hyperbaric oxygen preconditioning
Veltkamp et al.^19^	10	0	HBO reduces blood–brain barrier damage and edema after transient focal cerebral ischemia.	#3 EPR oximetry
Beynon et al.^20^	9	0.13	Delayed HBO is more effective than early prolonged normobaric hyperoxia in experimental focal cerebral ischemia.	#4 tPA
Eschenfelder et al.^21^	8	0.26	Neuroprotection by oxygen in acute transient focal cerebral ischemia is dose dependent and shows superiority of HBO.	#1 hyperbaric oxygen

EPR: Electron paramagnetic resonance; HBO: hyperbaric oxygen; HBOT: hyperbaric oxygen therapy; tPA: tissue plasminogen activator.

### Efficacy and application of hyperbaric oxygen in stroke treatment

In a rat stroke model, HBO had significant neuroprotective effects, with superior effects to those of the air control group and the normobaric oxygen group; specifically, HBO had a reduced infarct volume and markedly improved neurological function scores.^20^ The therapeutic effect exhibited a clear dose-dependent relationship.^21^

The therapeutic efficacy of HBO is primarily determined by the combined effects of treatment pressure and exposure duration. According to Henry’s law, the solubility of a gas in a liquid at a constant temperature is proportional to the partial pressure of the gas on the liquid surface.^41^ Therefore, the total oxygen dose is commonly employed in clinical practice to quantify HBO exposure intensity and is calculated via the following formula: total oxygen dose = treatment pressure × single-session duration × number of treatments.^42^ Importantly, the duration of HBOT is not positively correlated with outcome improvement, and prolonged continuous exposure may lead to oxygen toxicity and neurological impairment.^32^,^43^

The use of HBOT in stroke treatment remains controversial. According to the recommendations of the 10^th^ European Consensus Conference on Hyperbaric Medicine, HBOT may be considered a possible/optional measure for chronic stroke (Grade 3 recommendation, Level C evidence), whereas its use is not recommended for acute stroke (Grade 1 recommendation, Level C evidence).^44^ The timing of HBOT intervention appears to be a critical determinant of its therapeutic efficacy. Animal studies have demonstrated that HBOT can produce neuroprotective effects within 12 hours after stroke, which significantly reduce the infarct size.^32^,^36^ However, the application of HBO beyond 12 hours or at later timepoints may cause harm to patients.^23^,^45^ While current animal research confirms the value of early HBOT intervention (within 12 hours) for stroke, this optimal treatment window shows significant disparity with clinical reality.^36^ Thirty years ago, some clinical trials on HBOT in acute stroke patients failed to demonstrate therapeutic benefits.^33^,^34^,^35^ In contrast, numerous clinical studies on chronic-phase stroke have reported positive treatment outcomes.^13^,^25^,^29^,^30^

At present, there is no unified standard for the optimal timing and treatment course of HBOT in stroke patients. The effects of HBOT vary depending on the type of stroke (ischemic, hemorrhagic or embolic) and the time window after onset. In clinical practice, HBO is commonly combined with intravenous thrombolysis/endovascular thrombectomy (reperfusion therapy), neuroprotective agents, stem cell therapy and rehabilitation training. Based on the available clinical evidence, the recommended parameters of the treatment regimen are as follows: treatment pressure: 1.5–2.5 ATA; single treatment duration: 40–90 minutes; and adoption of an intermittent oxygen delivery strategy, with a total of 10–90 treatments.^13^,^24^,^25^,^26^,^27^,^28^,^29^,^30^,^31^,^32^,^33^,^34^,^35^ A multicenter trial (*n* = 565) demonstrated that HBOT at 1.5 ATA for 60 sessions is one of the optimal treatment options.^28^

HBOT is generally safe, and most adverse reactions are mild and reversible. Common adverse reactions include barotrauma (such as middle ear barotrauma,^13^,^25^,^32^,^33^ sinus barotrauma, and pulmonary barotrauma), oxygen toxicity, claustrophobia, seizures,^13^,^33^,^34^ anxiety, visual disturbances, and pneumothorax.

### Mechanisms of hyperbaric oxygen in the treatment of stroke

The core mechanism of HBOT lies in improving the tissue oxygen supply by increasing the oxygen partial pressure and dissolved oxygen content in the blood.^40^ Under physiological conditions, most oxygen in the blood binds to hemoglobin, whereas plasma-dissolved oxygen remains very low (~0.29 mL/dL blood, 0.3% v/v). Administering pure oxygen at > 1 ATA significantly increases plasma-dissolved oxygen in HBOT. For instance, at 1.5 ATA, the dissolved oxygen content can increase to 3.26% (v/v), and at 2.5 ATA, this proportion further increases to 5.6% (v/v). This increase in dissolved oxygen allows for more efficient diffusion of oxygen into ischemic tissues, thereby alleviating local hypoxia.^39^ This mechanism is particularly critical for patients with ischemic stroke. After stroke, the penumbra tissue surrounding the ischemic core retains a degree of reversibility. Timely administration of HBOT to increase the oxygen supply could help salvage these dying brain cells and improve clinical outcomes.^36^ Secondly, HBOT mitigates ischemia–reperfusion injury and protects brain tissue post-stroke by inhibiting free radical generation, reducing lipid peroxidation, and modulating inflammatory factor expression.^40^,^46^,^47^ Studies have shown that HBOT stimulates vascular endothelial growth factor expression, promotes neovascularization and improves brain microcirculation. Furthermore, HBOT activates neural stem cells, enhances axonal regeneration and synaptic remodeling, and accelerates neural functional recovery.^23^ HBOT can regulate the expression of apoptosis-related proteins (such as B-cell lymphoma 2),^15^ maintain mitochondrial membrane stability, inhibit ischemic brain cell apoptosis, and reduce brain injury.^48^

### Limitations

This review only searched the Web of Science Core Collection database, including a total of 231 articles, and did not cover other important databases, such as PubMed and Embase, which may have resulted in incomplete information. Future research should expand the search scope and further analyze research perspectives to ensure the comprehensiveness and representativeness of the literature.

### Conclusions and prospects

HBOT has significant potential for neuroprotection and functional recovery in stroke. However, its therapeutic efficacy, safety profile, and underlying mechanisms require further investigation. Future research should focus on the following: 1) conduct multicenter, large-sample randomized controlled trials to validate the efficacy and safety of HBOT; 2) integrate clinical and basic research to investigate the mechanisms of HBOT, particularly its roles in neuroprotection, neuroregeneration, and vascular repair, and to determine the optimal therapeutic time window; 3) establish standardized treatment protocols by developing unified therapeutic parameters, including pressure levels, session duration, and treatment frequency; and 4) explore the combined application of HBOT with adjunctive therapies (including pharmacotherapy, rehabilitation training, and stem cell-based neuroregeneration) to improve clinical outcomes in stroke patients. This study provides critical evidence for optimizing stroke treatment strategies, plays an important role in alleviating the burden of stroke-related diseases, and promotes the widespread clinical application of HBOT.

## Data Availability

*Not applicable.*
